# Interplay of Viral Infection, Host Cell Factors and Tumor Microenvironment in the Pathogenesis of Nasopharyngeal Carcinoma

**DOI:** 10.3390/cancers10040106

**Published:** 2018-04-04

**Authors:** Shaina Chor Mei Huang, Sai Wah Tsao, Chi Man Tsang

**Affiliations:** School of Biomedical Sciences, University of Hong Kong, Hong Kong SAR, HK, China; shainah@connect.hku.hk (S.C.M.H.); gswtsao@hku.hk (S.W.T.)

**Keywords:** nasopharyngeal carcinoma, Epstein-Barr virus, tumor microenvironment

## Abstract

Undifferentiated nasopharyngeal carcinoma (NPC) is strongly associated with Epstein-Barr virus (EBV) infection. In addition, heavy infiltration of leukocytes is a common characteristic of EBV-associated NPC. It has long been suggested that substantial and interactive impacts between cancer and stromal cells create a tumor microenvironment (TME) to promote tumorigenesis. The coexistence of tumor-infiltrating lymphocytes with EBV-infected NPC cells represents a distinct TME which supports immune evasion and cancer development from the early phase of EBV infection. Intracellularly, EBV-encoded viral products alter host cell signaling to facilitate tumor development and progression. Intercellularly, EBV-infected cancer cells communicate with stromal cells through secretion of cytokines and chemokines, or via release of tumor exosomes, to repress immune surveillance and enhance metastasis. Although high expression of miR-BARTs has been detected in NPC patients, contributions of these more recently discovered viral products to the establishment of TME are still vaguely defined. Further investigations are needed to delineate the mechanistic linkage of the interplay between viral and host factors, especially in relation to TME, which can be harnessed in future therapeutic strategies.

## 1. Introduction

Nasopharyngeal carcinoma (NPC) is a rare cancer in most parts of the world, but has a particularly high incidence in Southeast Asia and Southern China, especially in the Guangdong Province [1]. According to the World Health Organization (WHO) classification, NPC can be distinguished into two main subtypes: Non-keratinizing and keratinizing squamous cell carcinoma [2]. In Southern China, the non-keratinizing NPC is the predominant subtype and is closely associated with Epstein-Barr virus (EBV) infection [1,3,4].

The EBV is a ubiquitous virus that infects >90% of the global population [5,6,7,8]. EBV serology reveals a close association between NPC and EBV [8,9]. While latent EBV infection is rarely detected in normal nasopharyngeal epithelium, it is consistently detected in pre-invasive nasopharyngeal lesions as well as invasive NPC, suggesting that EBV infection is an early event in NPC development [10]. In addition to EBV infection, a familial history of NPC is another risk factor for the disease. Environmental risk factors including consumption of Cantonese-style preserved food such as salted fish, occupational exposures to formaldehyde, and phorbol ester contamination of water supplies in endemic areas have also been proposed [6,11,12]. It has been postulated that these environmental factors can induce DNA damage to cells and promote viral replication of EBV in latently infected B-cells. This may lead to increased rate of EBV infection of premalignant nasopharyngeal epithelium and contribute to the high incidence rate of NPC in endemic areas.

NPC cells demonstrate latency type II of EBV infection with the expression of a limited set of latent viral proteins, including the latent membrane proteins (LMP1 and LMP2A/B) and nuclear antigen (EBNA1) [13,14]. In addition, NPCs also express high levels of non-adenylated EBV-encoded small RNAs (EBERs 1 and 2). Abundant expression of microRNAs encoded from the BamHI-A region of the viral genome (miR-BARTs) is another distinctive feature of NPC [15,16,17]. There are many studies reporting how the EBV-encoded viral products interfere with host signaling to promote NPC pathogenesis, which will be summarized here.

The oncogenic property of EBV is supported by its ability to immortalize and transform primary B-cells in vitro [7,8,18]. While the EBV can efficiently infect, immortalize and transform B-lymphocytes [19,20], most epithelial cells are not susceptible to EBV infection in vitro [15,21]*.* Moreover, most EBV-infected NPC cell lines established in vitro readily lost their EBV genomes upon propagation, suggesting that EBV infection per se does not confer selective growth advantage to NPC cells propagated in culture [22,23]. However, EBV episomes are readily detected and maintained at high levels in NPC patients. This indicates EBV infection may confer growth advantage to NPC cells within the tumor microenvironment (TME) in vivo. The elucidation of the nature of this selective growth advantage will provide important insights to the role of EBV infection in NPC pathogenesis. There is growing evidence to support that EBV-infected NPC cells can secrete cytokines and exosomes containing viral products to modulate the function of stromal cells in TME to facilitate NPC progression and evade host immune attacks.

## 2. Alterations of Intracellular Cell Signaling in EBV-Infected NPC Cells

EBV activates multiple cellular signaling pathways in NPC cells [24]. The modulation of host cell signaling plays important roles in suppressing senescence and apoptosis, promoting cell growth and facilitating malignant transformation of infected cells. The roles of viral products expressed in the latency II program including LMP1/LMP2, EBNA1 and miR-BARTs in promoting NPC development and progression will be reviewed here [15,17,23]. 

### 2.1. The Tumorigenic Property of EBV-Encoded Latent Proteins

#### 2.1.1. LMP1 Alters Multiple Cell Signaling Pathway and Reprograms Cell Metabolism in NPC Cells

The LMP1 has been well documented as a viral oncoprotein in EBV-associated malignancies [25]. Distinct LMP1 variants are associated with NPC in the endemic region [26,27]. While the explanation of the prevalence of specific LMP1 variants in endemic NPC remains to be sought, there is evidence suggesting that immune selection mechanism may be involved [28]. Compared to the B95.8-LMP1 strain (derived from infectious mononucleosis), key amino acid changes were observed in the more prevalent LMP1 variants in NPC patients. These changes may potentially alter the human leukocyte antigen (HLA)-restricted epitopes of different LMP1 variants [28]. This is likely to affect the immunogenicity of the LMP1-expressing NPC cells and render the cytotoxic T-cell activity less effective. A phenotypic distinction between epithelial cells expressing B95.8-LMP1 and 2117-LMP1 (a prevalent LMP1 strain associated with NPC in HK) has also been demonstrated [26,29]. The B95.8-LMP1 was also shown to be more cytotoxic than 2117-LMP1 in cells. This could be the reason for the selection of 2117-LMP1 as the dominant LMP1 variant in EBV-associated NPC. LMP1 is a potent activator of NF-κB signaling through the two activating regions in its carboxy-terminal cytoplasmic domain, CTAR1 and CTAR2 [30]. The NPC-derived 2117-LMP1 is also more potent than the B95.8-LMP1 in activating nuclear factor-κB (NF-κB) signaling [26]. 

The oncogenic roles of LMP1 have been studied extensively and reviewed in details [7,8,15,23,25,31]. Interestingly, expression of LMP1 is common in high-grade dysplastic lesions in the nasopharyngeal epithelium infected with EBV and has been implicated in facilitating the expansion of EBV-infected cell populations at early stage of NPC development [23]. An earlier study showed that expression of LMP1 inhibited epithelial differentiation by upregulating the expression of cluster of differentiation (CD) 40 and intercellular adhesion molecule-1 (ICAM-1) [32]. LMP1 suppressed the formation of cross-linked envelopes and the terminal differentiation of infected epithelial cells. Besides, LMP1 was shown to induce the expression of Id1 through the activation of NF-κB signaling [33]. Since Id1 is a potent negative transcriptional regulator of p16^INK4a^, LMP1 may help to suppress the onset of replicative senescence in epithelial cells by inhibiting the CDK4/p16^INK4a^ regulatory pathway.

Furthermore, a relatively novel role of LMP1 in reprogramming the cellular metabolism is emerging. It was found to enhance aerobic glycolysis to promote NPC pathogenesis [34,35]. Aerobic glycolysis, also known as the Warburg Effect, is the reprogramming of cellular metabolic pathways in cancer cells to accommodate the cell growth and proliferation. The metabolic shift towards aerobic glycolysis involves increased expression of glycolytic enzymes, upregulation of glucose transporters, and inhibition of mitochondrial metabolism [36]. LMP1 could upregulate hexokinase 2 (HK2) via activation of c-Myc, upregulation of HK2 could also promote aerobic glycolysis and facilitate growth of tumor cells by blocking apoptosis under hypoxic conditions [35]. More recently, our laboratory has demonstrated that LMP1 can activate mTORC1 signaling to accelerate aerobic glycolysis and enhance NPC malignancy via the mTORC1/NF-κB activation of Glut-1 signaling cascade [34]. The fact that LMP1 contributes to the reprogramming of cancer metabolism further strengthens the role of EBV infection in adapting to alterations of host intracellular factors to promote NPC pathogenesis.

#### 2.1.2. Multiple Latent EBV-Encoded Proteins (LMP1, LMP2A, EBNA1) Promote Invasive and Metastatic Properties of NPC Cells

Metastasis is common in NPC and is the major cause of treatment failure in patients [37,38]. Metastasis is a highly complicated and multi-step process during which cancer cells need to pass through a sequence of events from shedding into circulation for transportation, surviving a tough environment within the blood vessels and capillaries, arresting in the new destination organ, to finally initiating and maintaining the growth and survival of the metastatic tumor [39]. The epithelial-mesenchymal transition (EMT) is regarded as a critical mechanism to enhance cell motility and initiate metastatic progression [40]. Multiple EBV-encoded viral products including LMP1, LMP2A and EBNA1 are involved in promoting NPC metastasis.

LMP1 is able to promote NPC metastasis via different mechanisms. It has been shown that LMP1 can upregulate the formation of focal adhesion (FA) complexes to promote epithelial cell migration [41]. LMP1 expression is positively correlated with the expression of Snail (a common EMT marker) in NPC tissues, and LMP1 expression in human nasopharyngeal cells was shown to induce Snail expression, thus promoting EMT [42]. As a potent activator of NF-κB, LMP1 promotes the transcription of tumor necrosis factor α-induced protein 2 (TNFAIP2). TNFAIP2 associates with actin to form actin-based membrane protrusions and contributes to LMP1-induced cell motility [43]. LMP1 is found to be associated with activation of matrix metalloproteinase 9 (MMP9), which can degrade type IV collagen in the basement membrane to facilitate cancer invasion. Correlation of MMP9 expression and lymph node metastasis was also reported [44]. Apart from directly modulating host signaling cascades, LMP1 can also promote metastasis by altering host miRNAs levels. LMP1 can induce pro-metastatic miR-10b to promote NPC metastasis [45]. Furthermore, LMP1 could also downregulate the tumor suppressor miR-204 to enhance the activity of Cdc42, which mediates cellular invasive properties such as focal complex formation, integrin localization, and MMP expression [46]. A recent study also demonstrated that through the suppression of miR-203 in an NF-κB-dependent manner, LMP1 can induce activation of cadherin 6 (CDH6) to act as the synergic node of multiple pathways to promote EMT [47]. Runt-related transcription factor 2 (RUNX2) can also be upregulated by LMP1 in similar manner, and high levels of CDH6 and RUNX2 have been identified in NPC tissues from patients with bone metastasis [47].

LMP2A expression can also be detected in NPC cells [48,49], and interestingly, it is mainly localized at the invasive tumor front [50]. It was also shown that expression of LMP2A can induce the expression of EMT-like molecular alteration in NPC cells and increase the stem cell-like population, hence promoting the cellular invasiveness and metastatic abilities of NPC cells [50]. In NPC biopsy samples, the LMP2A level has been correlated with the level of pro-metastatic cellular protein integrin-α-6 (ITGα6) [51]. Similar to LMP1, LMP2A can also increase MMP9 levels, but through the enhanced production of transcription factor Fra-1 via the ERK1/2 pathway [52]. Another way that LMP2A is involved in promoting EMT in NPC is via induction of the PI3K/Akt/mTOR pathway to phosphorylate 4EBP1. The activated 4EBP1-eIF4E axis can then upregulate expression of metastatic tumor antigen 1 (MTA1), which promotes EMT via the Wnt1 pathway and β-catenin activation [53]. A more recent study has shown that EBV-encoded viral proteins including LMP2A can markedly upregulate sphingosine kinase 1 (SPHK1), which can produce sphingosine-1 phosphate (S1P) [54]. And via the SPHK1/S1P/S1PR axis, Akt can be activated to promote cell migration in EBV-associated NPC [54].

Another EBV-encoded viral product, EBNA1, is expressed in all forms of latency in EBV-infected cells and is required to maintain EBV genome copy numbers in EBV-associated NPC [31,55]. Multiple oncogenic properties of EBNA1 are reported and reviewed in detail elsewhere [14,55]. EBNA1 is also implicated in contributing to the metastatic process of NPC. EBNA1 can enhance the activity of the AP-1 transcription factor and hence increase the expression of factors such as vascular endothelial growth factor (VEGF) and hypoxia-inducible factor 1 alpha (HIF-1α), and has been shown to promote angiogenesis in vitro [56]. Expression of EBNA1 has also been found to be associated with lymph node metastasis and able to upregulate the EMT mediators zinc finger E-box binding homeobox 1 (ZEB1) and ZEB2 [57]. The aforementioned study also showed that EBNA1 can upregulate S1P to promote NPC cell migration [54].

### 2.2. The Multiple Roles of EBV-Encoded BART microRNAs in NPC

#### 2.2.1. The EBV-Encoded BART microRNAs Contribute to Viral Latency Maintenance and Promote Cell Survival

Viral proteins such as LMPs are highly immunogenic and their expressions in NPC biopsies are often low and heterogeneous. In contrast, another set of non-immunogenic EBV-encoded products—BART-microRNAs (miR-BARTs)—is consistently detected in high abundance in primary NPC tumors and the established NPC cell line, C666-1 [16,58,59,60,61,62,63], EBV-encoded miRNAs have been found to constitute a considerable portion (commonly > 30%) of total miRNAs in NPC samples and EBV-positive NPC cell line, C666-1 [60,61]. The miR-BARTs are expressed at high levels in NPC while miR-BHRF expression (commonly expressed in EBV-infected B cells) is much lower [58,59,60]. Although the expression of individual miR-BARTs is shown to be highly variable, the overall miR-BART expression profiles are generally consistent across NPC biopsy samples, NPC xenografts and the EBV positive C666-1 NPC cells [16,58,61]. Interestingly, while abundant expression of miR-BARTs is commonly observed in NPC biopsies, it is hard to maintain the miR-BARTs at high levels in in vitro and ex vivo systems [62,63]. This once again suggests that the specific TME in the in vivo environment is essential in the regulation of expression of miR-BARTs.

These miR-BARTs have been reported to directly and indirectly regulate viral gene expressions to maintain viral latency and to promote cell survival. An early study using EBV-infected gastric carcinoma cell lines reported that miR-BART20-5p can directly target lytic genes BZLF1 (Zta) and BRLF1 (Rta) to stabilize viral latency [64]. While in NPC-related studies, a DNA double-strand break repair gene—ataxia telangiectasia mutated (ATM), was found to be involved in promoting viral lytic replication in nasopharyngeal epithelial cells [65]. Consistent with this, a recent study has demonstrated that four miR-BARTs (miR-BART5-5p, miR-BART7-3p, miR-BART9-3p, miR-BART14-3p) co-operatively suppress ATM activity to inhibit BZLF1 expressions in viral lytic replication [66]. These have demonstrated the potentials of miR-BARTs in maintaining EV viral latency in infected cells.

Besides, miR-BARTs also modulates LMP1 levels in NPCs to promote cell survival. Heterogeneous levels of LMP1 have been detected in NPC biopsy samples [7]. LMP1 could promote tumorigenicity at low expression level [67]. In contrast, elevated levels of LMP1 are often cytotoxic in epithelial cells and sensitize NPC cells to chemotherapeutic agents and inhibit cell growth [68,69]. Cluster 1 miR-BARTs have been shown to negatively regulate LMP1 to optimize its levels of expression in NPCs to promote cell survival [70]. 

#### 2.2.2. miR-BARTs Support NPC Progression by Targeting Growth- and Apoptosis-Related Factors

Apart from maintaining viral latency, numerous studies have supported the ability of miR-BARTs to promote NPC growth and proliferation while inhibiting apoptosis in EBV-associated NPC cells. One of the abundantly-expressed miR-BARTs, miR-BART7, was able to increase the rate of proliferation significantly when overexpressed in EBV-negative NPC cell lines [71]. Silencing miR-BART7-3p was shown to reduce NPC cell growth in vitro and in vivo [72]. Another EBV miRNA, miR-BART3-5p, has also revealed an ability to simulate the cell proliferation rate by targeting 3’-untranslated region (UTR) of deleted in cancer 1 (DICE1) [73]. The DICE1 is known to have a tumor suppressor gene function and inhibits colony formation in tumor cells [74]. The expression of DICE1 and miR-BART3-5p has been shown to be inversely correlated, and miR-BART3-5p was able to stimulate cell proliferation by counteracting the function of DICE1 [73]. A more recent report further demonstrated the ability of miR-BARTs to potentiate tumor growth in an in vivo system. Upon introduction of tumor cells into mice models, the expression of all miR-BARTs was upregulated in tumors developed in vivo [62]. The miR-BART-expressing tumors were more aggressive, incurring a 10-fold higher tumor burden in immunocompromised mice compared with control tumors in vivo, and growing almost twice as fast as the control tumors [62].

While the mutation rate of canonical p53 is low in Chinese NPC [75,76], a study has shown that miR-BARTs have another way to target p53-related apoptotic pathways [77]. The miR-BART5 has been shown to target a pro-apoptotic protein, the p53 upregulated modulator of apoptosis (PUMA), which is a critical mediator of p53-dependent and -independent apoptosis pathways that can be induced by various stress stimuli. Notably, miR-BART5 is abundantly expressed in all NPC tumors whereas PUMA-β expression is undetectable in many NPC samples. The C666-1 NPC cells expressing lower levels of PUMA-β are also more resistant to apoptosis [77].

In 2011, Marquitz and colleagues discovered another pro-apoptotic target of miR-BARTs, the Bcl-2 interacting mediator of cell death (Bim), which belongs to the same BH3-only family as PUMA. Immunoblots and luciferase reporter assays showed that the combined expression of the Cluster I miR-BARTs was sufficient to downregulate Bim at the protein level, and addition of Cluster II miR-BARTs could further reduce the expression of Bim. Yet no individual miR-BART has been identified with potent suppressive effects on Bim. It has been suggested that the negative regulation of Bim requires binding of the entire 3’-UTR by multiple miRNAs [78]. Proceeding from the Marquitz findings, Cullen’s group conducted a larger-scale study to determine if the highly expressed miR-BARTs in EBV-transformed epithelial cells have anti-apoptotic functions. Through multiple assays they identified a series of at least seven pro-apoptotic mRNA targets of miR-BARTs. Target knockdown assays demonstrated causal relationships between the mRNA targets and the observed reduction in apoptosis, including miR-BART3 targeting DICE1 and CASZ1a, miR-BART6 targeting OCT1, miR-BART16 targeting CREBBP and SH2B3, and miR-BART22 targeting PAK2 and TP53INP1 [79]. Except for DICE1 which had been previously reported [73], the group concluded that the remaining six were newly identified targets [79]. A more recent study presented another pro-apoptotic target of miR-BARTs: the central executioner of apoptosis, Caspase 3 (CASP3). Several BART microRNAs were shown to be able to repress CASP3 protein, among which miR-BART22 showed the most significant repressive effects [80].

#### 2.2.3. miR-BARTs Promote Invasive and Metastatic Properties of NPC Cells

In addition to their function in promoting cell survival, a number of miR-BARTs have demonstrated the ability to promote EMT and have pro-metastatic effects. For instance, miR-BART9 was reported to have no effect on tumor growth or proliferation, but could promote mesenchymal-like characteristics in NPC cells [81]. Apparently, miR-BART9 could specifically target and downregulate E-cadherin, which led to a redistribution of β-catenin [82]. This induced nuclear translocation of β-catenin triggered NPC cells to adopt a more motile and mesenchymal-like morphology. The ectopic expression of miR-BART9 in NPC cells was also able to upregulate the expression of multiple MMPs; and the cells also harbored lower levels of α-catenin (an epithelial marker) but a higher expression of vimentin (a mesenchymal marker) [81]. Other than targeting E-cadherin, miR-BART7-3p is able to induce EMT by targeting PTEN. miR-BART7-3p can regulate PI3K/Akt/GSK-3β and lead to the expression and relocalization of two important EMT-associated molecules: Snail and β-catenin [83,84]. Another study showed that miR-BART1 could also inhibit the expression of PTEN and was able to increase cell migration in vitro, and transplantation of these NPC cells into nude mice showed increased liver and lymph node metastases [85]. miR-BART1 induces tumor metastasis by activating PTEN-dependent pathways, and this effect was additive when both miR-BART1-3p and miR-BART1-5p were expressed [85]. There is also verification that miR-BART10-3p can upregulate the expression of β-catenin and Snail by targeting the β-transducin repeat-containing (BTRC) gene [86]. The BTRC gene encodes β-transducin repeat-containing E3 ubiquitin protein ligase (βTrCP), which recognizes β-catenin and Snail as degradation substrates. By inhibiting βTrCP-mediated ubiquitination, β-catenin and Snail are able to accumulate in NPC cells. MiR-BART10-3p can reduce the expression of epithelial markers while increasing the expression of mesenchymal markers, indicating the ability to promote EMT and metastasis [85].

Research has also shown that rather than individual miR-BARTs, all cluster 2 miR-BARTs can cooperate to downregulate the N-myc downstream regulated gene 1 (NDRG1) [87]. NDRG1 is expressed at low levels in B cells but at high levels in primary epithelial cells, acting as an epithelial cell-specific metastasis suppressor [87]. NDRG1 is shown to be endogenously downregulated in C666-1, which naturally expresses high levels of miR-BARTs [16,87].

## 3. Intercellular Interaction of EBV-Infected NPC Cells with Stromal Cells in TME

### 3.1. Cell Types Present in the TME of NPC

There are many reports supporting an important role of TME in NPC pathogenesis. The TME has cellular components and non-cellular ECM (extracellular matrix). In this review, we will focus our discussion in the composition, recruitment and involvement of cellular components of TME in immune suppression, growth and invasive properties of NPC.

A major characteristic of the NPC-TME is the presence of significant population of CD45^+^ tumor-infiltrating lymphocytes (TILs) in the NPC tumor stroma [88]. While the prognostic value of the massive infiltration of leucocytes remains to be determined, it has been demonstrated as a common feature of NPC primary tumors but less common in metastatic lesions [89]. The presence of infiltrating leucocytes may be more critical for the development of primary NPC. Advanced and metastatic NPC may be less dependent on these infiltrating leucocytes. The majority of lymphocyte infiltrates in NPC biopsies is CD3^+^ T-lymphocytes. Although the CD4^+^:CD8^+^ ratios vary across NPC specimens, together they generally comprise over 50% of the infiltrating lymphocytes in NPC [90]. Small subsets of B-lymphocytes, monocytes and macrophages, natural killer (NK) cells, and neutrophils are also detected in the TME of NPC [88,90]. The recruitment and activity of infiltrated leucocytes are believed to be determined by the concentration of different cytokines and chemokines present in TME. Increased levels of interleukin (IL)-6, IL-8, IL-10, and interferon (IFN)-γ and decreased levels of IL-2 are generally detected in NPC patients and biopsies and may be involved in the recruitment and activity of these infiltrating leucocytes [91,92,93,94]. In NPC, the role of the infiltrating leucocytes as pro- or anti-tumorigenic may be dependent on the real-time compositions and balances of the cytokine/chemokine profiles as well as the ratios of different immune cells present in the TME. The situations are further complicated by heterogeneous expression of latent EBV gene products in NPC cells and the disrupted latency in small subsets of NPC cells [23,95]. Nevertheless, great effort has been invested in studying the role of host-tumor interactions in establishing the dynamics of infiltrating leucocytes and their contribution to the immune evasion of EBV-infected NPC cells [88].

### 3.2. Recruitment of TME Cells

NPC cells and EBV-encoded viral products play important roles in triggering the release of various pro-inflammatory cytokines to promote the accumulation and homing of lymphocytes and other immune cells to TME. NPC cells express IL-18 [96] and CXCL-10 [97]. IL-18 and CXCL-10 may activate and induce IFN-γ production by CXCR3^+^ T-cells which in turn may stimulate CD68^+^ macrophages to produce IL-12 and IL-18, which can reciprocally stimulate IFN-γ production from T-cells and NK cells. IFN-γ can also enhance CXCL10 production by malignant epithelial cells [96,97], forming positive regulatory loops to promote inflammation and leukocyte infiltration. The EBV-encoded LMP1 can also induce CXCL-10 expression via NF-κB signaling [97].

Disrupted latency could be detected in small subsets of NPC cells with the expression of the EBV lytic transactivator, Zta [95]. The Zta can bind to the IL-8 promoter and induce IL-8 expression from the NPC cells [98]. IL-8 is known to be a potent chemoattractant for neutrophils, which are suggested to be the first immune cells invoked and recruited into the inflamed tissues [99,100,101]. Zta was also shown to manipulate neighboring monocytes to release IL-10 into the TME [102]. Zta can upregulate two immune-modulators in NPC cells: granulocyte-macrophage colony-stimulating factor (GM-CSF) and prostaglandin E2 (PGE_2_). The release of GM-CSF and PGE_2_ into the TME has a synergic effect in enhancing IL-10 production from monocytes [102]. The IL-10 has long been known as a potent immunosuppressive cytokine that can effectively inhibit the cytotoxic function of activated CD8^+^ cells to promote survival of EBV-infected NPC cells [103]. Activated monocytes produce two potent chemokines, CCL2 and CCL3, to attract various leukocytes into TME [104].

The EBV-encoded LMP1 has been reported to upregulate multiple cytokines and promote leukocyte infiltration into tumor sites [105,106,107]. Endogenous LMP1 in NPC cells can upregulate IL-8, macrophage inflammatory protein (MIP)-1α and MIP-1β to attract various types of leucocyte infiltration [106]. IL-8 can attract peripheral neutrophils and CXCR1^+^CD8^+^ T-cells; MIP-1α can recruit CD8^+^ T-cells and monocytes; and MIP-1β can recruit CD4^+^ and regulatory (T_reg_) T-cells [106]. As described earlier, LMP1 plays a role in mediating aerobic glycolysis in NPC [34]. A recent study showed that the recruitment and expansion of myeloid-derived suppressor cells (MDSCs) in NPC TME can be induced by LMP1-mediated glycolysis [105]. LMP1 mediates aerobic glycolysis through GLUT1 stabilization. The metabolic reprogramming of NPC by LMP1 alters cellular signaling and results in increased cyclooxygenase (COX)-2 expression, phospho-p65 and Nod-like receptor family protein 3 (NLRP3) inflammasomes. This gives rise to the increased release of IL-1β, IL-6, and GM-CSF into the TME and eventually leads to MDSC induction and expansion in TME [105].

Another group of consistently expressed EBV-encoded products, EBERs, also plays a role in constructing the TME. EBERs can induce inflammation in NPC via toll-like receptor 3 (TLR3) [108]. EBER activates the TLR3 pathway, and results in the production of inflammatory cytokines via NF-κB signaling. The recruitment and activation of macrophages induced by the inflammatory cytokines leads to development of TME to support tumor growth. Interestingly, a positive loop between EBER and LMP1 via NF-κB has also been demonstrated to reinforce the role of the inflammation-to-oncogenesis transition in NPC development [108].

The majority of the leucocytes infiltrating NPC comprises T-lymphocytes, among which the subsets of T-helper (Th)-1, Th-2, Th-17, and T_reg_ and cytotoxic T-cells (CTLs) play different roles in the TME. Compared with healthy controls, fewer circulating CD3^+^ CD45RA^+^naïve (T_naïve_) and CD4^+^ CD25^−^convention (T_conv_) T-cells were found [94]; a significantly higher percentage of CD25^+^ T_reg_ cells from both the CD4^+^ and CD8^+^ lineages were found in PBMCs and TILs from NPC patients; and IL-17-producing (CD8^+^ T_17_) cells were detected at lower levels in NPC patients than in healthy controls [94,109]. The CD8^+^ T_reg_ cells were found to be secreting high levels of IFN-γ and IL-10 but low levels of IL-2 and IL-17, while the CD8^+^ T_17_ produced high levels of IFN-γ and IL-17, intermediate levels of IL-10, and low levels of IL-2 [109]. The CD8^+^T_reg_ cells were shown to be able to inhibit CD4^+^ T_naïve_ cell proliferation through cell–cell contact in vitro [109]. It is noteworthy that naïve and memory T_reg_ cells express CCR4 and CCR6 on their cell surfaces, which show strong chemotactic responsiveness to corresponding chemokines CCL22 and CCL20, respectively [110,111]. High levels of CCL20 (MIP-3α) are reported in NPC biopsies and patient serum [91,109], which may be responsible for the chemotaxis of T_reg_ towards TME. In Hodgkin’s lymphoma the expression of EBNA1 has been shown to upregulate the production of CCL20 and induce the migration of T_reg_ cells [112], but this has yet to be confirmed in NPCs. In addition to the direct secretion of chemokines into the TME, it has been reported that malignant NPC cells can release exosomes for the transport of products into the TME [17,113]. NPC-derived exosomes in the TME can promote the chemotaxis of CD4^+^ CD25^+^ FoxP3^+^ T_reg_ cells [114]. A complex composition of infiltrated leucocytes in TME and their reciprocal interaction with EBV-infected NPC is emerging. Other stromal cells including activated fibroblasts and endothelial cells are also present in TME. Elucidation of their interactive relationship and roles in the development of NPC are future challenges.

### 3.3. Role of TME Cells and Viral Factors in Immune Evasion

In NPC, massive leucocytes infiltration appears not to be involved in elimination of malignant cells, but instead facilitating tumor development [88]. In patients with the undifferentiated NPC type, abnormal T-cell activity has been detected. CD4^+^ T-cells in the peripheral blood of patients were defective in IL-2 production while showing a moderate increase in IL-10 production, which is consistent with a dysregulated Th-1/Th-2 balance [115]. This may also contribute to the IL-2^lo^ IL-10^hi^ environment reported in NPC [91,92,93,94]. CD38/C1.7^+^ activated CD8^+^ T-cells predominate in the peripheral blood of patients and their secretion of IFN-γ and perforin was impaired, which may lead to inefficient antiviral and antitumor responses in NPC [115]. The IL-2^lo^ IL-10^hi^ environment may also contribute to the expansion of T-lymphocytes with impaired anti-tumor activity. A higher percentage of TILs were found to exhibit CD4^+^ CD25^+^ surface markers, which are designated to become T_reg_ cells. TILs expanded in IL-2^lo^ conditions lacked cytotoxicity activity hence they were unable to lyze autologous EBV-infected targets and failed to produce IFN-γ [94]. Interestingly, it has been reported that overall immunocompetence is preserved in NPC patients as EBV-specific CTLs can be reactivated from the patient’s blood. The activated CTLs can still traffic to tumor sites but are selectively rendered non-functional at the NPC tumor site [94]. Overcoming this immune suppression at TME at NPC site may offer effective treatment options for NPC.

The IL-10 appears to be an important immunosuppressive cytokine that helps malignant NPC cells to evade immune regulation. IL-10 may reduce antigen-specific T-cell proliferation by downregulating the class II MHC antigen on the antigen-presenting cells and by inhibiting the activity of CTLs and IL-2 production from T-helper cells [116]. The importance of IL-10 in mediating immune evasion may also be indicated by the fact that BCRF1, an EBV-encoded protein induced by lytic infection, is strongly homologous to IL-10 [117]. It has been coined as viral IL-10 (vIL-10) as it is functionally similar to endogenous IL-10 and is reported to exhibit immunosuppressive activity [118,119]. EBV-encoded viral products EBER and LMP1 have been significantly associated with IL-10 expression in NPC biopsy samples, and the upregulation of IL-10 appears to be associated with Fas-L production in NPC cells [120]. Upregulated production of Fas-L (CD95-L) may trigger T-cell apoptosis via crosslinking with Fas (CD95) expressed on the T-cells, triggering activation-induced cell death (AICD) in T-cells, hence protecting tumor cells [121,122]. Interestingly, NPC cells also express Fas and may also enter into AICD after binding with Fas-L [123,124]. Yet NPC cells may avoid AICD by consistently expressing abundant levels of CD40 which can bind to the CD40-L (CD154) produced by infiltrating T-cells to rescue NPC cells from AICD [123]. The LMP1 which has been shown to be a functional homologue of CD40 may play a similar role to rescue NPC cells from AICD [125].

In addition to the aforementioned functions, LMP1 may also play a role in inducing T-cell apoptosis by upregulating programmed death-lignand1 (PD-L1) expression on the NPC cell surface [126]. Cross-linking of PD-L1 on tumor cells and PD-1 on T-cells can suppress T-cell attack [127]. Upon EBV infection, LMP1 can mediate the upregulation of PD-L1 by activating STAT3, AP-1, and NF-κB signaling. IFN-γ has also been shown to induce PD-L1 transcription via JAK-STAT signaling in NPC cells [126]. While high levels of IFN-γ can help to recruit leukocytes to the TME, IFN-γ also exhibits growth-inhibitory effects. Resistance to such inhibitory effects may be supported by EBV viral factors: EBERs can avert the blockade of protein synthesis induced by IFNs [128]; LMP2s can enhance turnover in the intracellular pool of IFN-receptors via endosomal-mediated degradation and thus attenuate the inhibitory effects of IFN-γ on NPC cells [129].

### 3.4. The Role of TME in Promoting NPC Metastasis

As discussed earlier, NPC is a highly metastatic cancer, and lymph node metastases are common at diagnosis [37,38]. There are evidence suggesting that EBV-infected NPC cells interacts with TME components to facilitate metastasis. An increased presence of FoxP3^+^ T_reg_ cells and CD68^+^ tumor-associated macrophages (TAMs) has been found in EBV-positive NPC specimens and associated with poor prognosis [130,131]. Both cell types are also shown to interact with NPC cells to promote metastasis. Receptor activator of nuclear factor-kappa B (RANK) signaling is known to play a critical role in the metastasis of various cancers [132,133]. T_reg_ cells are also reported to be pro-metastatic by producing ligands of RANK, and thus triggering the migration of epithelial cancer cells that express RANK [132,133]. A recent study has shown that NPC metastases express high levels of RANK, despite unavailable results in primary NPC tumors [134]. Along with the evidence of higher circulating T_reg_ cells in NPC patients [88], it is likely that T_reg_ cells can produce RANKL to promote the migration of primary NPC cells via RANK-RANKL signaling.

Another recent study has revealed an interacting loop between NPC cells and TAMs in driving NPC metastasis [135]. EBV-associated NPC cells can produce VEGF and GM-CSF to attract monocytes to a tumor site and induce TAM-lineage differentiation, respectively. TAMs are potent producers of CCL18, which can further enhance NF-κB signaling in NPC cells, heighten the release of VEGF and GM-CSF, and induce EMT, forming a forward-feeding loop to drive NPC metastasis [135]. In vivo mice studies have also clearly shown that such an interplay between NPC and TAMs induces EMT in humanized but not in immunodeficient NOC/SCID mice [135]. This further demonstrates the significance of TME in NPC oncogenesis.

### 3.5. Exosome-Modulation of TME for Immune Evasion and Metastasis

Exosomes released by EBV-infected NPC cells may also play a role in immune evasion. A study on NPC exosomes (NPC-Exo) has shown that they contain viral product LMP1 and immunomodulatory protein galectin-9 [136]. LMP1 can induce broad anergic effects on immune cells such as the inhibition of T-cell proliferation, NK cytotoxicity, and IFN-γ release [137], while galectin-9 can suppress Th1 activity [138]. LMP1 has been shown to inhibit T-cell proliferation more efficiently than galectin-9 does [136]. NPC-Exo containing LMP1 demonstrated more potent effects in inhibiting the proliferation of CD4^+^CD25^−^ T_conv_ cells, thus suppressing the expansion of functional helper T-cells in the TME [114]. In the same study, NPC-Exo also promoted the conversion of T_conv_ into T_reg_, further suppressing the anti-tumor responses of leukocyte infiltrate. Compared to healthy donor-derived exosomes, NPC-Exo more efficiently induced the expansion of the T_reg_ population, which was also shown to function in producing immunosuppressive cytokines and suppressing PBMC proliferation [114].

While various EBV-encoded viral products such as LMP1, EBNA1, and EBERs have shown important roles in modulating the TME and immune evasion, the role of BART transcript products in intercellular communication within the TME is emerging. miR-BARTs have also been detected in NPC patient plasma samples and in NPC-Exo [139]. However, the detailed transportation mechanisms and fates of these exosomal microRNAs are yet to be discovered. In terms of biological functioning, microRNAs are potent regulators of protein expression. It would be interesting to determine whether individual miR-BARTs or their clusters can be transported into stromal cells and modulate the TME to contribute to NPC progression.

Exosome-mediated metastasis has already been implicated in several human malignancies, as tumor-derived exosomes were reported to carry pro-EMT factors such as TGF-β, HIF-1α, and β-catenin, which can enhance the metastatic properties of recipient cells [140]. In the context of NPC, LMP1 has been shown to play a role in upregulating HIF-1α levels in LMP1^+^-NPC-Exo [141]. Through the incorporation of exosomal HIF-1α into neighboring NPC cells, the transcriptional-active HIF-1α can trigger an EMT program and increase N-cadherin levels while decreasing E-cadherin levels in cells. The exosome-treated cells have been shown to exhibit more invasive phenotypes and activity [141]. NPC-Exo has also been shown to contain MMP-13, which are known to digest the ECM and facilitate the protrusion of invasive cancer cells [142]. In addition, NPC-Exo carries the oncogenic HS1-associated protein X-1 (HAX-1), which can accelerate NPC tumor growth and angiogenesis [143]. Compared to exosomes derived from nasopharyngeal epithelial cell lines (NP69 and NP460), the EBV^+^NPC cell line (C666-1)-derived exosomes were shown to contain upregulated levels of the pro-angiogenic protein ICAM-1 and the CD44 variant isoform 5 (CD44v5), as well as downregulated levels of the angio-inhibitory protein thrombospondin-1 [144]. C666-1-derived exosomes were also able to promote the migration and invasiveness of HUVECs and alter the expression patterns of angiogenic proteins in HUVECs, further demonstrating the potential of NPC-Exo to influence neighboring cells to promote metastasis [144]. Given the hints that LMP1 plays a part in determining the content and release of NPC-Exo, such as increased levels of HIF-1α in exosomes [141], it would be interesting to know whether specific viral products also play a part in regulating the packaging of NPC-Exo in different situations.

## 4. Conclusions and Future Perspectives

The association of EBV infection with undifferentiated NPC has been well established. Several EBV-encoded viral genes including LMP1, LMP2A/B, EBNA1 have been implicated in NPC pathogenesis by altering host cell signaling. Interestingly, EBV-infected NPC cells do not exhibit selective growth advantages in vitro as evidenced by the common loss of EBV episomes in NPC cells propagated in culture. Yet, EBV infection is universally associated with NPC in patients. Heavy infiltration of leukocytes is a common characteristic of EBV-associated NPC. Together with the other stromal cells, they form a unique TME which has been postulated to play an important role to support growth and progression of NPC cells in patients. More and more evidences have emerged to support a role of EBV infection in immune evasion and anti-apoptosis which may give growth advantages of EBV-infected cells during NPC pathogenesis. However, the potential mechanisms of miR-BARTs in repressing immune surveillance and promoting metastasis of NPC remain to be fully elucidated. The essential role of TME of NPC in the maintenance of persistent EBV infection and regulation of viral gene expression is emerging. Many studies have reported the influence of EBV-encoded gene products on stromal cells in the TME of NPC through secretion of cytokines and chemokines. The release of tumor exosomes from EBV-infected NPC cells may be an important means to modulate properties and behaviors of stromal cells in TME to impact on NPC pathogenesis and progression. Further investigation is needed to clearly define the components of NPC-Exo and the mechanisms that modify the TME. Revelation of the exosomal transportation of viral factors in circulations may also provide new opportunities to develop biomarkers for the prognosis of EBV-associated NPC, especially in predicting metastatic potentials. Taken together, delineation of the intricate interactions of EBV-infected NPC with stromal cells in the TME will be one of the future directions in the research of EBV and NPC, and this may reveal novel targets for effective treatment of NPC in patients.
